# Supplementary material for: HIVprotI: an integrated web based platform for prediction and design of HIV proteins inhibitors
Source: J Cheminform. 2018 Mar 9;10:12. doi: 10.1186/s13321-018-0266-y (PMC5845081; doi:10.1186/s13321-018-0266-y)
Supplement: Supplementary file 2 — Additional file 2. Source code of HIVProtI web server. [file 13321_2018_266_MOESM2_ESM.zip › HIVprotI_Source-code/hivprotI/weka-3-6-6/doc/weka/gui/visualize/PostscriptGraphics.html]

PostscriptGraphics


---


|  |  |  |
| --- | --- | --- |
| **Overview** \| **Package** \| **Class** \| **Tree** \| **Deprecated** \| **Index** \| **Help** \| **Weka's home** | |  |
| **PREV CLASS**   **NEXT CLASS** | **FRAMES**    **NO FRAMES**     **All Classes** |  |
| SUMMARY: NESTED \| FIELD \| CONSTR \| METHOD | DETAIL: FIELD \| CONSTR \| METHOD |  |


---


## weka.gui.visualize Class PostscriptGraphics

```
java.lang.Object
  java.awt.Graphics
      java.awt.Graphics2D
          weka.gui.visualize.PostscriptGraphics
```

---

``` public class PostscriptGraphics extends java.awt.Graphics2D ```

The PostscriptGraphics class extends the Graphics2D class to
produce an encapsulated postscript file rather than on-screen display.

Currently only a small (but useful) subset of Graphics methods have been
implemented.
To handle the ability to Clone a Graphics object, the graphics state of the
eps is set from the graphics state of the local PostscriptGraphics before output.
To use, create a PostscriptGraphics object, and pass it to the PaintComponent
method of a JComponent.

If necessary additional font replacements can be inserted, since some fonts
might be displayed incorrectly.

**Version:**
:   $Revision: 7059 $

**Author:**
:   Dale Fletcher (dale@cs.waikato.ac.nz), FracPete (fracpete at waikato dot ac dot nz)

**See Also:**: `addPSFontReplacement(String, String)`, `m_PSFontReplacement`

---

**Constructor Summary**

`PostscriptGraphics(int width,
int height,
java.io.OutputStream os)`
  
          Constructor
Creates a new PostscriptGraphics object, given dimensions and
output file.


| **Method Summary** | |
| --- | --- |
| `static void` | `addPSFontReplacement(java.lang.String replace, java.lang.String with)`             adds the PS font name to replace and its replacement in the replacement hashtable |
| `void` | `addRenderingHints(java.util.Map m)` |
| `void` | `clearRect(int x, int y, int width, int height)`             Draw a filled rectangle with the background color. |
| `void` | `clip(java.awt.Shape s)` |
| `void` | `clipRect(int x, int y, int width, int height)`             Not implemented |
| `void` | `copyArea(int x, int y, int width, int height, int dx, int dy)`             Not implemented |
| `java.awt.Graphics` | `create()`             Clone a PostscriptGraphics object |
| `void` | `dispose()`             Not implemented |
| `void` | `draw(java.awt.Shape s)` |
| `void` | `draw3DRect(int x, int y, int width, int height, boolean raised)`             Draw an outlined rectangle with 3D effect in current pen color. |
| `void` | `drawArc(int x, int y, int width, int height, int startAngle, int arcAngle)`             Not implemented |
| `void` | `drawBytes(byte[] data, int offset, int length, int x, int y)`             simply calls drawString(String,int,int) |
| `void` | `drawChars(char[] data, int offset, int length, int x, int y)`             simply calls drawString(String,int,int) |
| `void` | `drawGlyphVector(java.awt.font.GlyphVector gv, float f1, float f2)` |
| `void` | `drawImage(java.awt.image.BufferedImage bi, java.awt.image.BufferedImageOp bio, int i1, int i2)` |
| `boolean` | `drawImage(java.awt.Image im, java.awt.geom.AffineTransform at, java.awt.image.ImageObserver io)` |
| `boolean` | `drawImage(java.awt.Image img, int x, int y, java.awt.Color bgcolor, java.awt.image.ImageObserver observer)`             calls drawImage(Image,int,int,int,int,Color,ImageObserver) |
| `boolean` | `drawImage(java.awt.Image img, int x, int y, java.awt.image.ImageObserver observer)`             calls drawImage(Image,int,int,Color,ImageObserver) with Color.WHITE as background color |
| `boolean` | `drawImage(java.awt.Image img, int x, int y, int width, int height, java.awt.Color bgcolor, java.awt.image.ImageObserver observer)`             PS see http://astronomy.swin.edu.au/~pbourke/geomformats/postscript/ Java http://show.docjava.com:8086/book/cgij/doc/ip/graphics/SimpleImageFrame.java.html |
| `boolean` | `drawImage(java.awt.Image img, int x, int y, int width, int height, java.awt.image.ImageObserver observer)`             calls drawImage(Image,int,int,int,int,Color,ImageObserver) with the color WHITE as background |
| `boolean` | `drawImage(java.awt.Image img, int dx1, int dy1, int dx2, int dy2, int sx1, int sy1, int sx2, int sy2, java.awt.Color bgcolor, java.awt.image.ImageObserver observer)`             Not implemented |
| `boolean` | `drawImage(java.awt.Image img, int dx1, int dy1, int dx2, int dy2, int sx1, int sy1, int sx2, int sy2, java.awt.image.ImageObserver observer)`             calls drawImage(Image,int,int,int,int,int,int,int,int,Color,ImageObserver) with Color.WHITE as background color |
| `void` | `drawLine(int x1, int y1, int x2, int y2)`             Draw a line in current pen color. |
| `void` | `drawOval(int x, int y, int width, int height)`             Draw an Oval outline in current pen color. |
| `void` | `drawPolygon(int[] xPoints, int[] yPoints, int nPoints)`             Not implemented |
| `void` | `drawPolyline(int[] xPoints, int[] yPoints, int nPoints)`             Not implemented |
| `void` | `drawRect(int x, int y, int width, int height)`             Draw an outlined rectangle in current pen color. |
| `void` | `drawRenderableImage(java.awt.image.renderable.RenderableImage ri, java.awt.geom.AffineTransform at)` |
| `void` | `drawRenderedImage(java.awt.image.RenderedImage ri, java.awt.geom.AffineTransform af)` |
| `void` | `drawRoundRect(int x, int y, int width, int height, int arcWidth, int arcHeight)`             Not implemented |
| `void` | `drawString(java.text.AttributedCharacterIterator aci, float f1, float f2)` |
| `void` | `drawString(java.text.AttributedCharacterIterator iterator, int x, int y)`             Not implemented |
| `void` | `drawString(java.lang.String str, float x, float y)` |
| `void` | `drawString(java.lang.String str, int x, int y)`             Draw text in current pen color. |
| `void` | `fill(java.awt.Shape s)` |
| `void` | `fill3DRect(int x, int y, int width, int height, boolean raised)`             Draw a filled rectangle with 3D effect in current pen color. |
| `void` | `fillArc(int x, int y, int width, int height, int startAngle, int arcAngle)`             Not implemented |
| `void` | `fillOval(int x, int y, int width, int height)`             Draw a filled Oval in current pen color. |
| `void` | `fillPolygon(int[] xPoints, int[] yPoints, int nPoints)`             Not implemented |
| `void` | `fillPolygon(java.awt.Polygon p)`             Not implemented |
| `void` | `fillRect(int x, int y, int width, int height)`             Draw a filled rectangle in current pen color. |
| `void` | `fillRoundRect(int x, int y, int width, int height, int arcWidth, int arcHeight)`             Not implemented |
| `void` | `finalize()`             Not implemented |
| `void` | `finished()`             Finalizes output file. |
| `java.awt.Color` | `getBackground()` |
| `java.awt.Shape` | `getClip()`             Not implemented |
| `java.awt.Rectangle` | `getClipBounds()`             This returns the full current drawing area |
| `java.awt.Rectangle` | `getClipBounds(java.awt.Rectangle r)`             This returns the full current drawing area |
| `java.awt.Rectangle` | `getClipRect()`             Not implemented |
| `java.awt.Color` | `getColor()`             Get current pen color. |
| `java.awt.Composite` | `getComposite()` |
| `java.awt.GraphicsConfiguration` | `getDeviceConfiguration()` |
| `java.awt.Font` | `getFont()`             Get current font. |
| `java.awt.FontMetrics` | `getFontMetrics(java.awt.Font f)`             Get Font metrics |
| `java.awt.font.FontRenderContext` | `getFontRenderContext()`             START overridden Graphics2D methods |
| `java.awt.Paint` | `getPaint()` |
| `java.lang.Object` | `getRenderingHint(java.awt.RenderingHints.Key key)` |
| `java.awt.RenderingHints` | `getRenderingHints()` |
| `java.awt.Stroke` | `getStroke()` |
| `java.awt.geom.AffineTransform` | `getTransform()` |
| `boolean` | `hit(java.awt.Rectangle r, java.awt.Shape s, boolean onstroke)` |
| `void` | `rotate(double d1)` |
| `void` | `rotate(double d1, double d2, double d3)` |
| `void` | `scale(double d1, double d2)` |
| `void` | `setBackground(java.awt.Color c)` |
| `void` | `setClip(int x, int y, int width, int height)`             Not implemented |
| `void` | `setClip(java.awt.Shape clip)`             Not implemented |
| `void` | `setColor(java.awt.Color c)`             Set current pen color. |
| `void` | `setComposite(java.awt.Composite c)` |
| `void` | `setFont(java.awt.Font font)`             Set current font. |
| `void` | `setPaint(java.awt.Paint p)` |
| `void` | `setPaintMode()`             Not implemented |
| `void` | `setRenderingHint(java.awt.RenderingHints.Key key, java.lang.Object o)` |
| `void` | `setRenderingHints(java.util.Map m)` |
| `void` | `setStroke(java.awt.Stroke s)` |
| `void` | `setTransform(java.awt.geom.AffineTransform at)` |
| `void` | `setXORMode(java.awt.Color c1)`             Not implemented |
| `void` | `shear(double d1, double d2)` |
| `void` | `transform(java.awt.geom.AffineTransform at)` |
| `void` | `translate(double d1, double d2)` |
| `void` | `translate(int x, int y)`             Translates the origin of the graphics context to the point (x, y) in the current coordinate system. |

**Methods inherited from class java.awt.Graphics**

`create, drawPolygon, getFontMetrics, hitClip, toString`

**Methods inherited from class java.lang.Object**

`equals, getClass, hashCode, notify, notifyAll, wait, wait, wait`

**Constructor Detail**

### PostscriptGraphics

```
public PostscriptGraphics(int width,
                          int height,
                          java.io.OutputStream os)
```

:   Constructor
    Creates a new PostscriptGraphics object, given dimensions and
    output file.

    **Parameters:**: `width` - The width of eps in points.: `height` - The height of eps in points.: `os` - File to send postscript to.


**Method Detail**

### finished

```
public void finished()
```

:   Finalizes output file.

---


### addPSFontReplacement

```
public static void addPSFontReplacement(java.lang.String replace,
                                        java.lang.String with)
```

:   adds the PS font name to replace and its replacement in the replacement
    hashtable

    :   **Parameters:**: `replace` - the PS font name to replace: `with` - the PS font name to replace the font with

---


### clearRect

```
public void clearRect(int x,
                      int y,
                      int width,
                      int height)
```

:   Draw a filled rectangle with the background color.

    :   **Specified by:**: `clearRect` in class `java.awt.Graphics`
    :   **Parameters:**: `x` - starting x coord: `y` - starting y coord: `width` - rectangle width: `height` - rectangle height

---


### clipRect

```
public void clipRect(int x,
                     int y,
                     int width,
                     int height)
```

:   Not implemented

    :   **Specified by:**: `clipRect` in class `java.awt.Graphics`

---


### copyArea

```
public void copyArea(int x,
                     int y,
                     int width,
                     int height,
                     int dx,
                     int dy)
```

:   Not implemented

    :   **Specified by:**: `copyArea` in class `java.awt.Graphics`

---


### create

```
public java.awt.Graphics create()
```

:   Clone a PostscriptGraphics object

    :   **Specified by:**: `create` in class `java.awt.Graphics`

---


### dispose

```
public void dispose()
```

:   Not implemented

    :   **Specified by:**: `dispose` in class `java.awt.Graphics`

---


### draw3DRect

```
public void draw3DRect(int x,
                       int y,
                       int width,
                       int height,
                       boolean raised)
```

:   Draw an outlined rectangle with 3D effect in current pen color.
    (Current implementation: draw simple outlined rectangle)

    :   **Overrides:**: `draw3DRect` in class `java.awt.Graphics2D`
    :   **Parameters:**: `x` - starting x coord: `y` - starting y coord: `width` - rectangle width: `height` - rectangle height: `raised` - True: appear raised, False: appear etched

---


### drawArc

```
public void drawArc(int x,
                    int y,
                    int width,
                    int height,
                    int startAngle,
                    int arcAngle)
```

:   Not implemented

    :   **Specified by:**: `drawArc` in class `java.awt.Graphics`

---


### drawBytes

```
public void drawBytes(byte[] data,
                      int offset,
                      int length,
                      int x,
                      int y)
```

:   simply calls drawString(String,int,int)

    :   **Overrides:**: `drawBytes` in class `java.awt.Graphics`
    :   **See Also:**: `drawString(String,int,int)`

---


### drawChars

```
public void drawChars(char[] data,
                      int offset,
                      int length,
                      int x,
                      int y)
```

:   simply calls drawString(String,int,int)

    :   **Overrides:**: `drawChars` in class `java.awt.Graphics`
    :   **See Also:**: `drawString(String,int,int)`

---


### drawImage

```
public boolean drawImage(java.awt.Image img,
                         int x,
                         int y,
                         java.awt.Color bgcolor,
                         java.awt.image.ImageObserver observer)
```

:   calls drawImage(Image,int,int,int,int,Color,ImageObserver)

    :   **Specified by:**: `drawImage` in class `java.awt.Graphics`
    :   **See Also:**: `drawImage(Image,int,int,int,int,Color,ImageObserver)`

---


### drawImage

```
public boolean drawImage(java.awt.Image img,
                         int x,
                         int y,
                         java.awt.image.ImageObserver observer)
```

:   calls drawImage(Image,int,int,Color,ImageObserver) with Color.WHITE as
    background color

    :   **Specified by:**: `drawImage` in class `java.awt.Graphics`
    :   **See Also:**: `drawImage(Image,int,int,Color,ImageObserver)`, `Color.WHITE`

---


### drawImage

```
public boolean drawImage(java.awt.Image img,
                         int x,
                         int y,
                         int width,
                         int height,
                         java.awt.Color bgcolor,
                         java.awt.image.ImageObserver observer)
```

:   PS see http://astronomy.swin.edu.au/~pbourke/geomformats/postscript/
    Java http://show.docjava.com:8086/book/cgij/doc/ip/graphics/SimpleImageFrame.java.html

    :   **Specified by:**: `drawImage` in class `java.awt.Graphics`

---


### drawImage

```
public boolean drawImage(java.awt.Image img,
                         int x,
                         int y,
                         int width,
                         int height,
                         java.awt.image.ImageObserver observer)
```

:   calls drawImage(Image,int,int,int,int,Color,ImageObserver) with the color
    WHITE as background

    :   **Specified by:**: `drawImage` in class `java.awt.Graphics`
    :   **See Also:**: `drawImage(Image,int,int,int,int,Color,ImageObserver)`, `Color.WHITE`

---


### drawImage

```
public boolean drawImage(java.awt.Image img,
                         int dx1,
                         int dy1,
                         int dx2,
                         int dy2,
                         int sx1,
                         int sy1,
                         int sx2,
                         int sy2,
                         java.awt.Color bgcolor,
                         java.awt.image.ImageObserver observer)
```

:   Not implemented

    :   **Specified by:**: `drawImage` in class `java.awt.Graphics`

---


### drawImage

```
public boolean drawImage(java.awt.Image img,
                         int dx1,
                         int dy1,
                         int dx2,
                         int dy2,
                         int sx1,
                         int sy1,
                         int sx2,
                         int sy2,
                         java.awt.image.ImageObserver observer)
```

:   calls drawImage(Image,int,int,int,int,int,int,int,int,Color,ImageObserver)
    with Color.WHITE as background color

    :   **Specified by:**: `drawImage` in class `java.awt.Graphics`
    :   **See Also:**: `drawImage(Image,int,int,int,int,int,int,int,int,Color,ImageObserver)`

---


### drawLine

```
public void drawLine(int x1,
                     int y1,
                     int x2,
                     int y2)
```

:   Draw a line in current pen color.

    :   **Specified by:**: `drawLine` in class `java.awt.Graphics`
    :   **Parameters:**: `x1` - starting x coord: `y1` - starting y coord: `x2` - ending x coord: `y2` - ending y coord

---


### drawOval

```
public void drawOval(int x,
                     int y,
                     int width,
                     int height)
```

:   Draw an Oval outline in current pen color.

    :   **Specified by:**: `drawOval` in class `java.awt.Graphics`
    :   **Parameters:**: `x` - x-axis center of oval: `y` - y-axis center of oval: `width` - oval width: `height` - oval height

---


### drawPolygon

```
public void drawPolygon(int[] xPoints,
                        int[] yPoints,
                        int nPoints)
```

:   Not implemented

    :   **Specified by:**: `drawPolygon` in class `java.awt.Graphics`

---


### drawPolyline

```
public void drawPolyline(int[] xPoints,
                         int[] yPoints,
                         int nPoints)
```

:   Not implemented

    :   **Specified by:**: `drawPolyline` in class `java.awt.Graphics`

---


### drawRect

```
public void drawRect(int x,
                     int y,
                     int width,
                     int height)
```

:   Draw an outlined rectangle in current pen color.

    :   **Overrides:**: `drawRect` in class `java.awt.Graphics`
    :   **Parameters:**: `x` - starting x coord: `y` - starting y coord: `width` - rectangle width: `height` - rectangle height

---


### drawRoundRect

```
public void drawRoundRect(int x,
                          int y,
                          int width,
                          int height,
                          int arcWidth,
                          int arcHeight)
```

:   Not implemented

    :   **Specified by:**: `drawRoundRect` in class `java.awt.Graphics`

---


### drawString

```
public void drawString(java.text.AttributedCharacterIterator iterator,
                       int x,
                       int y)
```

:   Not implemented

    :   **Specified by:**: `drawString` in class `java.awt.Graphics2D`

---


### drawString

```
public void drawString(java.lang.String str,
                       int x,
                       int y)
```

:   Draw text in current pen color.

    :   **Specified by:**: `drawString` in class `java.awt.Graphics2D`
    :   **Parameters:**: `str` - Text to output: `x` - starting x coord: `y` - starting y coord

---


### fill3DRect

```
public void fill3DRect(int x,
                       int y,
                       int width,
                       int height,
                       boolean raised)
```

:   Draw a filled rectangle with 3D effect in current pen color.
    (Current implementation: draw simple filled rectangle)

    :   **Overrides:**: `fill3DRect` in class `java.awt.Graphics2D`
    :   **Parameters:**: `x` - starting x coord: `y` - starting y coord: `width` - rectangle width: `height` - rectangle height: `raised` - True: appear raised, False: appear etched

---


### fillArc

```
public void fillArc(int x,
                    int y,
                    int width,
                    int height,
                    int startAngle,
                    int arcAngle)
```

:   Not implemented

    :   **Specified by:**: `fillArc` in class `java.awt.Graphics`

---


### fillOval

```
public void fillOval(int x,
                     int y,
                     int width,
                     int height)
```

:   Draw a filled Oval in current pen color.

    :   **Specified by:**: `fillOval` in class `java.awt.Graphics`
    :   **Parameters:**: `x` - x-axis center of oval: `y` - y-axis center of oval: `width` - oval width: `height` - oval height

---


### fillPolygon

```
public void fillPolygon(int[] xPoints,
                        int[] yPoints,
                        int nPoints)
```

:   Not implemented

    :   **Specified by:**: `fillPolygon` in class `java.awt.Graphics`

---


### fillPolygon

```
public void fillPolygon(java.awt.Polygon p)
```

:   Not implemented

    :   **Overrides:**: `fillPolygon` in class `java.awt.Graphics`

---


### fillRect

```
public void fillRect(int x,
                     int y,
                     int width,
                     int height)
```

:   Draw a filled rectangle in current pen color.

    :   **Specified by:**: `fillRect` in class `java.awt.Graphics`
    :   **Parameters:**: `x` - starting x coord: `y` - starting y coord: `width` - rectangle width: `height` - rectangle height

---


### fillRoundRect

```
public void fillRoundRect(int x,
                          int y,
                          int width,
                          int height,
                          int arcWidth,
                          int arcHeight)
```

:   Not implemented

    :   **Specified by:**: `fillRoundRect` in class `java.awt.Graphics`

---


### finalize

```
public void finalize()
```

:   Not implemented

    :   **Overrides:**: `finalize` in class `java.awt.Graphics`

---


### getClip

```
public java.awt.Shape getClip()
```

:   Not implemented

    :   **Specified by:**: `getClip` in class `java.awt.Graphics`

---


### getClipBounds

```
public java.awt.Rectangle getClipBounds()
```

:   This returns the full current drawing area

    :   **Specified by:**: `getClipBounds` in class `java.awt.Graphics`
    :   **Returns:**: full drawing area

---


### getClipBounds

```
public java.awt.Rectangle getClipBounds(java.awt.Rectangle r)
```

:   This returns the full current drawing area

    :   **Overrides:**: `getClipBounds` in class `java.awt.Graphics`
    :   **Returns:**: full drawing area

---


### getClipRect

```
public java.awt.Rectangle getClipRect()
```

:   Not implemented

    :   **Overrides:**: `getClipRect` in class `java.awt.Graphics`

---


### getColor

```
public java.awt.Color getColor()
```

:   Get current pen color.

    :   **Specified by:**: `getColor` in class `java.awt.Graphics`
    :   **Returns:**: current pen color.

---


### getFont

```
public java.awt.Font getFont()
```

:   Get current font.

    :   **Specified by:**: `getFont` in class `java.awt.Graphics`
    :   **Returns:**: current font.

---


### getFontMetrics

```
public java.awt.FontMetrics getFontMetrics(java.awt.Font f)
```

:   Get Font metrics

    :   **Specified by:**: `getFontMetrics` in class `java.awt.Graphics`
    :   **Parameters:**: `f` - Font **Returns:**: Font metrics.

---


### setClip

```
public void setClip(int x,
                    int y,
                    int width,
                    int height)
```

:   Not implemented

    :   **Specified by:**: `setClip` in class `java.awt.Graphics`

---


### setClip

```
public void setClip(java.awt.Shape clip)
```

:   Not implemented

    :   **Specified by:**: `setClip` in class `java.awt.Graphics`

---


### setColor

```
public void setColor(java.awt.Color c)
```

:   Set current pen color. Default to black if null.

    :   **Specified by:**: `setColor` in class `java.awt.Graphics`
    :   **Parameters:**: `c` - new pen color.

---


### setFont

```
public void setFont(java.awt.Font font)
```

:   Set current font. Default to Plain Courier 11 if null.

    :   **Specified by:**: `setFont` in class `java.awt.Graphics`
    :   **Parameters:**: `font` - new font.

---


### setPaintMode

```
public void setPaintMode()
```

:   Not implemented

    :   **Specified by:**: `setPaintMode` in class `java.awt.Graphics`

---


### setXORMode

```
public void setXORMode(java.awt.Color c1)
```

:   Not implemented

    :   **Specified by:**: `setXORMode` in class `java.awt.Graphics`

---


### translate

```
public void translate(int x,
                      int y)
```

:   Translates the origin of the graphics context to the point (x, y) in the
    current coordinate system. Modifies this graphics context so that its new
    origin corresponds to the point (x, y) in this graphics context's original
    coordinate system. All coordinates used in subsequent rendering operations
    on this graphics context will be relative to this new origin.

    :   **Specified by:**: `translate` in class `java.awt.Graphics2D`
    :   **Parameters:**: `x` - the x coordinate.: `y` - the y coordinate.

---


### getFontRenderContext

```
public java.awt.font.FontRenderContext getFontRenderContext()
```

:   START overridden Graphics2D methods

    :   **Specified by:**: `getFontRenderContext` in class `java.awt.Graphics2D`

---


### clip

```
public void clip(java.awt.Shape s)
```

:   **Specified by:**: `clip` in class `java.awt.Graphics2D`

---


### getStroke

```
public java.awt.Stroke getStroke()
```

:   **Specified by:**: `getStroke` in class `java.awt.Graphics2D`

---


### getBackground

```
public java.awt.Color getBackground()
```

:   **Specified by:**: `getBackground` in class `java.awt.Graphics2D`

---


### setBackground

```
public void setBackground(java.awt.Color c)
```

:   **Specified by:**: `setBackground` in class `java.awt.Graphics2D`

---


### getComposite

```
public java.awt.Composite getComposite()
```

:   **Specified by:**: `getComposite` in class `java.awt.Graphics2D`

---


### getPaint

```
public java.awt.Paint getPaint()
```

:   **Specified by:**: `getPaint` in class `java.awt.Graphics2D`

---


### getTransform

```
public java.awt.geom.AffineTransform getTransform()
```

:   **Specified by:**: `getTransform` in class `java.awt.Graphics2D`

---


### setTransform

```
public void setTransform(java.awt.geom.AffineTransform at)
```

:   **Specified by:**: `setTransform` in class `java.awt.Graphics2D`

---


### transform

```
public void transform(java.awt.geom.AffineTransform at)
```

:   **Specified by:**: `transform` in class `java.awt.Graphics2D`

---


### shear

```
public void shear(double d1,
                  double d2)
```

:   **Specified by:**: `shear` in class `java.awt.Graphics2D`

---


### scale

```
public void scale(double d1,
                  double d2)
```

:   **Specified by:**: `scale` in class `java.awt.Graphics2D`

---


### rotate

```
public void rotate(double d1,
                   double d2,
                   double d3)
```

:   **Specified by:**: `rotate` in class `java.awt.Graphics2D`

---


### rotate

```
public void rotate(double d1)
```

:   **Specified by:**: `rotate` in class `java.awt.Graphics2D`

---


### translate

```
public void translate(double d1,
                      double d2)
```

:   **Specified by:**: `translate` in class `java.awt.Graphics2D`

---


### getRenderingHints

```
public java.awt.RenderingHints getRenderingHints()
```

:   **Specified by:**: `getRenderingHints` in class `java.awt.Graphics2D`

---


### addRenderingHints

```
public void addRenderingHints(java.util.Map m)
```

:   **Specified by:**: `addRenderingHints` in class `java.awt.Graphics2D`

---


### setRenderingHints

```
public void setRenderingHints(java.util.Map m)
```

:   **Specified by:**: `setRenderingHints` in class `java.awt.Graphics2D`

---


### getRenderingHint

```
public java.lang.Object getRenderingHint(java.awt.RenderingHints.Key key)
```

:   **Specified by:**: `getRenderingHint` in class `java.awt.Graphics2D`

---


### setRenderingHint

```
public void setRenderingHint(java.awt.RenderingHints.Key key,
                             java.lang.Object o)
```

:   **Specified by:**: `setRenderingHint` in class `java.awt.Graphics2D`

---


### setStroke

```
public void setStroke(java.awt.Stroke s)
```

:   **Specified by:**: `setStroke` in class `java.awt.Graphics2D`

---


### setPaint

```
public void setPaint(java.awt.Paint p)
```

:   **Specified by:**: `setPaint` in class `java.awt.Graphics2D`

---


### setComposite

```
public void setComposite(java.awt.Composite c)
```

:   **Specified by:**: `setComposite` in class `java.awt.Graphics2D`

---


### getDeviceConfiguration

```
public java.awt.GraphicsConfiguration getDeviceConfiguration()
```

:   **Specified by:**: `getDeviceConfiguration` in class `java.awt.Graphics2D`

---


### hit

```
public boolean hit(java.awt.Rectangle r,
                   java.awt.Shape s,
                   boolean onstroke)
```

:   **Specified by:**: `hit` in class `java.awt.Graphics2D`

---


### fill

```
public void fill(java.awt.Shape s)
```

:   **Specified by:**: `fill` in class `java.awt.Graphics2D`

---


### drawGlyphVector

```
public void drawGlyphVector(java.awt.font.GlyphVector gv,
                            float f1,
                            float f2)
```

:   **Specified by:**: `drawGlyphVector` in class `java.awt.Graphics2D`

---


### drawString

```
public void drawString(java.text.AttributedCharacterIterator aci,
                       float f1,
                       float f2)
```

:   **Specified by:**: `drawString` in class `java.awt.Graphics2D`

---


### drawString

```
public void drawString(java.lang.String str,
                       float x,
                       float y)
```

:   **Specified by:**: `drawString` in class `java.awt.Graphics2D`

---


### drawRenderableImage

```
public void drawRenderableImage(java.awt.image.renderable.RenderableImage ri,
                                java.awt.geom.AffineTransform at)
```

:   **Specified by:**: `drawRenderableImage` in class `java.awt.Graphics2D`

---


### drawRenderedImage

```
public void drawRenderedImage(java.awt.image.RenderedImage ri,
                              java.awt.geom.AffineTransform af)
```

:   **Specified by:**: `drawRenderedImage` in class `java.awt.Graphics2D`

---


### drawImage

```
public void drawImage(java.awt.image.BufferedImage bi,
                      java.awt.image.BufferedImageOp bio,
                      int i1,
                      int i2)
```

:   **Specified by:**: `drawImage` in class `java.awt.Graphics2D`

---


### drawImage

```
public boolean drawImage(java.awt.Image im,
                         java.awt.geom.AffineTransform at,
                         java.awt.image.ImageObserver io)
```

:   **Specified by:**: `drawImage` in class `java.awt.Graphics2D`

---


### draw

```
public void draw(java.awt.Shape s)
```

:   **Specified by:**: `draw` in class `java.awt.Graphics2D`


---


|  |  |  |
| --- | --- | --- |
| **Overview** \| **Package** \| **Class** \| **Tree** \| **Deprecated** \| **Index** \| **Help** \| **Weka's home** | |  |
| **PREV CLASS**   **NEXT CLASS** | **FRAMES**    **NO FRAMES**     **All Classes** |  |
| SUMMARY: NESTED \| FIELD \| CONSTR \| METHOD | DETAIL: FIELD \| CONSTR \| METHOD |  |


---
